# Concurrent Validity and Feasibility of Short Tests Currently Used to Measure Early Childhood Development in Large Scale Studies

**DOI:** 10.1371/journal.pone.0160962

**Published:** 2016-08-22

**Authors:** Marta Rubio-Codina, M. Caridad Araujo, Orazio Attanasio, Pablo Muñoz, Sally Grantham-McGregor

**Affiliations:** 1 Social Protection and Health Division, Inter-American Development Bank, Washington, D.C., United States of America; 2 Centre for the Evaluation of Development Policies, Institute for Fiscal Studies, London, United Kingdom; 3 Department of Economics, University College London, London, United Kingdom; 4 École de Psychologie, Université Laval, Quebec, Canada; 5 Faculty of Population Health Sciences, Institute of Child Health, University College London, London, United Kingdom; Institute for Health & the Environment, UNITED STATES

## Abstract

In low- and middle-income countries (LIMCs), measuring early childhood development (ECD) with standard tests in large scale surveys and evaluations of interventions is difficult and expensive. Multi-dimensional screeners and single-domain tests (‘short tests’) are frequently used as alternatives. However, their validity in these circumstances is unknown. We examined the feasibility, reliability, and concurrent validity of three multi-dimensional screeners (Ages and Stages Questionnaires (ASQ-3), Denver Developmental Screening Test (Denver-II), Battelle Developmental Inventory screener (BDI-2)) and two single-domain tests (MacArthur-Bates Short-Forms (SFI and SFII), WHO Motor Milestones (WHO-Motor)) in 1,311 children 6–42 months in Bogota, Colombia. The scores were compared with those on the Bayley Scales of Infant and Toddler Development (Bayley-III), taken as the ‘gold standard’. The Bayley-III was given at a center by psychologists; whereas the short tests were administered in the home by interviewers, as in a survey setting. Findings indicated good internal validity of all short tests except the ASQ-3. The BDI-2 took long to administer and was expensive, while the single-domain tests were quickest and cheapest and the Denver-II and ASQ-3 were intermediate. Concurrent validity of the multi-dimensional tests’ cognitive, language, and fine motor scales with the corresponding Bayley-III scale was low below 19 months. However, it increased with age, becoming moderate-to-high over 30 months. In contrast, gross motor scales’ concurrence was high under 19 months and then decreased. Of the single-domain tests, the WHO-Motor had high validity with gross motor under 16 months, and the SFI and SFII expressive scales showed moderate correlations with language under 30 months. Overall, the Denver-II was the most feasible and valid multi-dimensional test and the ASQ-3 performed poorly under 31 months. By domain, gross motor development had the highest concurrence below 19 months, and language above. Predictive validity investigation is needed to further guide the choice of instruments for large scale studies.

## Introduction

Recent research demonstrates the importance of the early years to brain development, cognitive, language and socio-emotional development and, more generally, to human capital formation [1, 2]. Longitudinal studies show that poverty in early childhood has sustained effects on children’s development [3] and it is estimated that well over 200 million children under five years in low- and middle-income countries (LIMCs) are failing to reach their developmental potential [4]. Interventions in early childhood can have comprehensive benefits to life outcomes [5, 6, 7] and there is an increasing global commitment to implement such interventions at large scale in LIMCs in order to promote the development of disadvantaged children. The Sustainable Development Goals (SDGs), for example, include the aim that “all girls and boys have access to quality early childhood development, care and pre-primary education so that they are ready for primary education” by 2030 (SDG 4.2) [8].

The launching of early childhood development (ECD) interventions is nonetheless hindered by the lack of reliable and valid measures of child development that can be collected cost-effectively in large samples [9, 10]. Such measures are essential both to assess developmental levels of populations and to monitor and evaluate the effectiveness of interventions, which can inform the design of improved variants. The need for measures of ECD outcomes is particularly pressing for children under 3 years-of-age. Hence, there is an urgency to identify readily available valid and feasible methods to assess children’s development in large samples via household surveys (i.e. ‘at-scale’).

Multi-dimensional diagnostic tests such as the Bayley Scales of Infant Development [11, 12] are considered to be the ‘gold standard’ to measure the developmental levels of infants and toddlers [10, 13, 14]. Importantly, this test has shown sensitivity to differences in ECD outcomes due to interventions in diverse contexts [15, 16, 17]. However, test administration is time consuming and requires highly trained professionals working in controlled environments; test kits and administration fees are expensive; and identifying professional testers who can administer it in local languages is challenging. In addition, translation and adaptation to different languages and cultural contexts requires substantial technical skills, time, and financial resources. These reasons make the Bayley and similar diagnostic tests often unfeasible for use at-scale.

As an alternative, tests designed to screen for children at risk of delay or to assess specific developmental domains (e.g. language) are increasingly used in large scale surveys and impact evaluations [18, 19, 20]. Although not designed for this purpose and often not validated nor standardized locally, these tests are becoming popular alternatives since they are shorter, cheaper, and easier to administer. They can be administered by regular interviewers in the children’s homes, and often include a number of items collected by maternal report. Nonetheless, little is known about their validity when administered at-scale, not for screening, but to measure levels of child development across the range of developmental skills for either research purposes or to provide population-based assessments.

Two recent exceptions are the studies by Hamadani and colleagues in rural Bangladesh [21, 22]. The authors found moderate correlations between monthly maternal reports of age of attainment of motor milestones—primarily, walking and standing alone—and the Bayley-II Psychomotor Development Index (PDI) and low but significant associations with the Mental Development Index (MDI) at 18 months of age and with IQ at 5 years [21]. Similarly, a language test for children 12–18 months developed locally from the MacArthur-Bates Communicative Development Inventories [23] and administered by maternal report offered moderate concurrent validity with the Bayley-II MDI and acceptable predictive validity with IQ at age 5 years [22].

Interestingly, maternal reports of age of walking alone and language were as predictive of motor development or IQ at 64 months as the PDI and the MDI of the Bayley-II, respectively.

More recently, new multi-dimensional diagnostic tests for use in LMICs have become available for children 24 months and/or over [14, 24]. However, they do not cover children under 2 years of age and some continue to be too long for use at-scale [24].

The current study was therefore designed to investigate the extent to which a selection of multi-dimensional screeners and single-domain tests (‘short tests’ henceforth), were valid and feasible alternatives to diagnostic tests for the assessment of very young children at-scale. Specifically, we aimed to determine the administration time and cost, internal consistency and test-retest reliability, and concurrent validity of five short tests administered under survey conditions to measure the developmental levels of a population-based sample of children 6–42 months in Bogota, Colombia. The short tests were selected on the basis of their current use in large scale studies in the field, and its total number was limited to avoid tiring the child. We considered three multi-dimensional screeners—the Ages and Stages Questionnaires (third edition, ASQ-3) [25], the Denver Developmental Screening Test (second edition, Denver-II) [26, 27], and the Battelle Developmental Inventory screener (second edition, BDI-2) [28]—and two single-domain tests—the vocabulary checklists in the Short-Forms of the MacArthur-Bates Communicative Development Inventories I and II (SFI and SFII) [29, 30] and the World Health Organization gross motor milestones (WHO-Motor, WHO 2006) [31, 32]. The latter two tests share many similarities with those used in the above Bangladeshi studies [21, 22], and were included in addition to multi-dimensional screeners since they are quicker to administer.

To compute concurrent validity, children’s developmental scores on these short tests were compared to their scores on the Bayley Scales of Infant and Toddler Development (third edition, Bayley-III) [12]. As the ‘gold standard’, the Bayley-III was administered in ideal conditions—namely, at a center by trained psychologists. Nonetheless, and importantly to address the research question of interest, all short tests were administered under survey conditions—this is, in the children’s home by non-specialized albeit rigorously trained interviewers.

We investigated concurrent validity of the short tests with the Bayley-III by child’s age and developmental domain with a focus on cognitive, receptive and expressive language, and fine and gross motor development. Although we recognize socio-emotional development as an important developmental domain and we collected the scale, it was not analyzed. The Bayley-III uses the Greenspan Social-Emotional Growth Chart [33] for the measurement of socio-emotional development, which comprises only maternal report and is reasonably quick and easy to give. Moreover, the personal-social and adaptive scales of the short tests measure slightly different constructs from the socio-emotional scale of the Bayley-III, being more related to self-care and self-direction. Finally, only two subscales in the adaptive behavior questionnaire in the Bayley-III, which uses the Adaptive Behavior Assessment System (second edition, ABAS-II) [34], were collected on a subsample of children because of time constraints so they are not reported here either.

We hypothesized increased concurrent validity with age, as well as higher concurrence between scales measuring the same domain. However, scales measuring different developmental domains would also be correlated.

The study was not designed to establish the sensitivity or specificity of the screener tests in identifying high risk children. Furthermore, the number of children at risk of developmental delay in the sample was too small to allow carrying out such analyses. Rather, we were interested in examining the ability of the short tests to measure child development across the range of developmental levels in our study population, representative of low- and lower-middle income groups in a typical large city in Latin America. The aim was to identify feasible and easy-to-use readily available instruments for the assessment of very young children in large scale studies and in contexts different for those for which the tools were developed, thus guiding the choice of instruments for research purposes (for example, in program evaluations) and/or population-based assessments.

## Materials and Methods

### Participants

**Fig 1:**
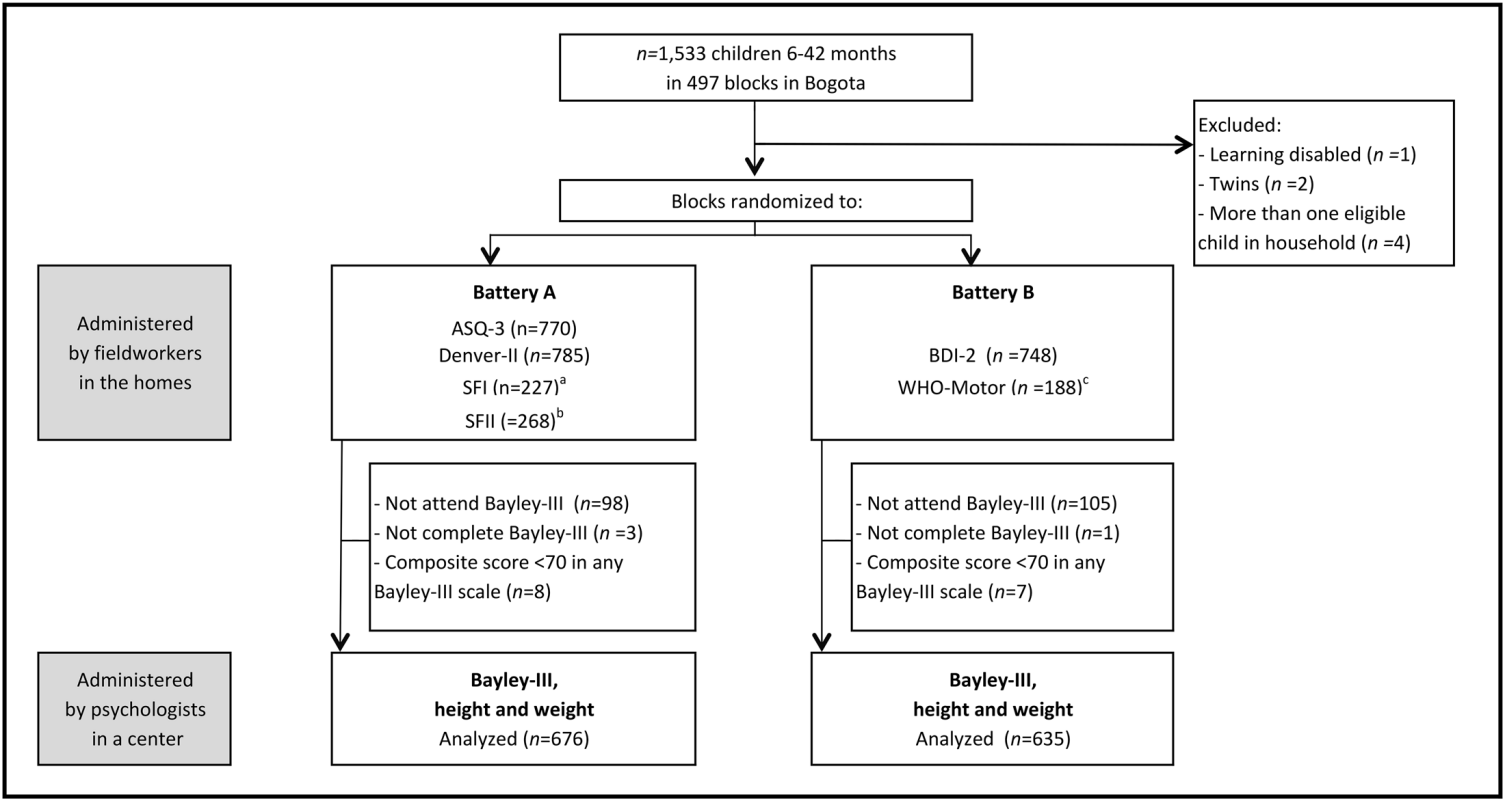
Flow Diagram of Study Participants and Study Design. ^a^Children 8–18 months. ^b^Children 19–30 months. ^c^Children 6–15 months.

Bogota is divided into six socio-economic strata, denominated ‘sectors’, based on location and quality of housing and infrastructure. Between March and August 2011, we enrolled 1,533 children aged 6–42 months randomly selected from the poorest three sectors and stratified by age and sector. These three sectors account for 85% of the city’s population and comprise low- and lower-middle-income households. Sector 4 (middle-income) was initially included but subsequently dropped due to high refusal rates to participate in the study. Nonetheless, the 12 children from this sector already tested were kept in the analysis. The Bayley-III is designed to assess children from birth to 42 months. One learning disabled child and a pair of twins were excluded (Fig 1). In households with more than one available child, one was randomly selected. Further details on the sample are provided elsewhere [35, 36].

### Procedures

To increase the number of tests examined, and minimize test weariness, children were randomly assigned to one of two batteries of short tests. Battery A included the ASQ-3, the Denver-II, and in children between 8 and 30 months the SFI or the SFII, depending on the child’s age. Battery B comprised the BDI-2 and in children 6–15 months the WHO-Motor. Both batteries took similar total amounts of time to be administered.

Non-specialized interviewers administered the short tests and a household survey in the children’s homes. The latter collected information on socio-economic background, which was used to construct a wealth index using principal component analysis of assets and housing as described in earlier work [35]. We also collected education for all household members and other individual characteristics, as well as the quality of the home environment using UNICEF’s Family Care Indicators (FCI) [37]. Specifically, we recorded, by observation, the number of books for adults, newspapers/magazines, and the toys the child usually played with by type; and by caregiver report, the play activities the child and an adult engaged in over the week prior to the survey. The short tests were administered in the order listed within the battery and after the first section of the household survey, once rapport with the caregiver had been established.

After 5 to 14 days, psychologists (testers henceforth), who were blind to children’s performance on the short tests, administered the Bayley-III at a nearby center.

All measurements took place in the presence of the main caregiver—the mother in 85–89% of the cases—who responded to test items when appropriate. Between 2.5 and 5% of the sessions, either in the home or the center, had to be rescheduled because the child was too sick or fussy to be tested.

Table 1 presents characteristics of the criterion test, the Bayley-III, and of the short tests to be validated, which are divided into multi-dimensional screeners and single-domain tests.

**Table 1:** Characteristics of the Bayley-III and the Short Tests.

	Age Range Test (mths)	Age Range Study Children (mths)	Number of items^a^	Time to administer (min)^b^	Cost (USD)^c^	Cronbachalpha	Test-retest ICC
**Criterion Measure**							
**Bayley-III**	0–42	6–42		(*n* = 36)	$1025 kit + $4.89 pc	(*n* = 1311)	(*n* = 20)
Cognition			21	83.2 (18.8)		0.97	0.96
Receptive Language			18			0.96	0.96
Expressive Language			16			0.96	0.98
Fine Motor			18			0.96	0.98
Gross Motor			16			0.97	0.98
**Multi-dimensional Tests for Validation**							
**ASQ-3**	1–66	6–42		(*n* = 32)	$275 kit	(*n* = 664)	(*n* = 12)
Problem Solving			6–9 (6.5)	19.7 (8.2)		0.60	0.80
Communication			6–9 (6.5)			0.68	0.92
Fine Motor			6–9 (6.4)			0.57	0.37
Gross Motor			6–9 (6.6)			0.70	0.90
Personal-Social			6–9 (6.4)			0.55	0.73
**Denver-II**	0–71	6–42		(*n* = 32)	$200 kit + $0.45 pc	(*n* = 658)	(*n* = 12)
Language			10	27 (10.5)		0.93	0.93
Fine Motor-Adaptive			9			0.91	0.83
Gross Motor			9			0.90	0.53
Personal-Social			9			0.91	0.49
**BDI-2 (Battelle)**	0–83	6–42		(*n* = 30)	$405.70 kit + $3.08 pc	(*n* = 635)	(*n* = 11)
Cognitive			9	59 (13.0)		0.79	0.92
Communication			9			0.89	0.94
Motor			9			0.88	0.98
Personal-Social			10			0.84	0.71
Adaptive Skills			9			0.84	0.90
**Single-domain Tests for Validation**							
**SFI (MacArthur)**	8–18	8–18		(*n* = 8)	$90 kit + $1 pc	(*n* = 192)	(*n* = 12)
Receptive Language			104	8.6 (1.9)		0.97	0.99
Expressive Language			104			0.92	
**SFII (MacArthur)**	19–30	19–30		(*n* = 10)		(*n* = 226)	
Expressive Language			100	8.2 (3.3)		0.981	NA
**WHO-Motor**	4–24	6–15		(*n* = 9)	free	(*n* = 152)	(*n* = 11)
Gross Motor			6	6 (2.7)		0.86	0.80

^a^ Average number of items assessed per study participant. The original ASQ-3 only has 6 items per scale.

^b^ Data are Mean (Standard Deviation) in minutes, as recorded by the trainer during supervision activities.

^c^ pc is 'per child' administration fee.

Test kits include record forms in packages of 100 for the Denver-II, packages of 30 for the BDI-2, and packages of 25 for the rest of the short tests and the Bayley-III. Information on costs last consulted on line in March 2016. The Denver-II is now freely available. The WHO-Motor was not available in Spanish; and only parts of the BDI-2 were available. The Bayley-III is available in Spanish since mid-2015. All other tests and manuals were available in Spanish.

#### Criterion Measure

The Bayley-III was used as the ‘gold standard’ and the cognitive, receptive and expressive language, and fine and gross motor scales were administered to all children in the sample. These scales are entirely assessed by direct observation of the child’s performance in a series of tester-administered items, arranged in increasing order of difficulty. Basal and ceiling rules determine the number of items to administer to each child.

#### Short Tests for Validation

The ASQ-3, Denver-II and BDI-2 are multi-dimensional screeners and cover the entire age range. The ASQ-3 comprises age-specific caregiver-completed questionnaires, each assessing five developmental domains. Given the low education levels of some caregivers, items were given by interview and were only administered directly to the child if the caregiver could not provide an answer. Whenever a child attained the maximum score in a scale, we modified the administration and gave the first three new items from subsequent questionnaires. This increased the variability of developmental abilities captured by the test and reduced the number of children on the test ceiling by 10.5–15.5% to levels of 1.7–4.8%, depending on the domain. A similar adaptation has been used elsewhere [18]. The Denver-II assesses four domains mainly by direct administration, although up to 39% of the items were collected by caregiver report, most of which are in the personal-social and language scales. Whilst there is no cognitive scale per se, the fine motor-adaptive scale combines both fine motor and cognitive items. Basal and ceiling rules around an age line establish the number of items administered to each child per scale. The BDI-2 screener measures five domains, the motor scale combining fine and gross motor items. Items can be administered by direct administration to the child, by observation of the child during the test and by caregiver report. As in the Bayley-III, items in the Denver-II and BDI-2 are arranged in increasing order of difficulty. All three screeners combine receptive and expressive language into one communication/language scale.

The remaining short tests, the SFs and the WHO-Motor are single-domain and have a limited age-range. The SFI and SFII are vocabulary checklists administered by caregiver report. The SFI assesses receptive and expressive language—the words the child ‘understands’ and ‘understands and says’, respectively—for children 8–18 months. The SFII only measures expressive language—the number of words the child ‘says’—for children 19–30 months. The WHO-Motor includes six milestones, all given by direct administration, to assess the gross motor development of children 6–18 months. Analysis was however limited to children 6–15.9 months, since 91.9% of those older, attained all milestones.

#### Preparation for Testing

The Bayley-III was translated and back-translated to ensure linguistic and functional equivalence. All short tests in battery A were available in Spanish. In battery B, the BDI-2 manual, and the WHO-Motor report forms and manual required translation. Piloting of all tests suggested minor wording and phrasing modifications to either the translated or official Spanish versions in order to reflect Colombian Spanish. Similarly, a few images had to be contextualized.

Six female psychology graduates were trained on the Bayley-III for six weeks; and eight female interviewers, with no university education and no prior experience testing children, were trained on the short tests in either battery A or battery B for seven weeks. Trainers had master degrees in Psychology. Practice testing for testers and interviewers occurred in pairs and continued until inter-observer reliabilities between trainee-trainer were >0.9 (intra-class correlations, *ICCs*) on each test. Each tester/interviewer carried out around 20 practice administrations on each test that she was trained on. During data collection, 5% of assessments were observed by the trainer and corrective feedback was given when appropriate. The agreement between tester and trainer scores during these tests was high (*ICC*s mean = 0.95), ensuring high data quality.

### Statistical Analyses

For all tests, scales were administered and scored independently, producing test-domain-specific assessments. For each scale, continuous raw scores were constructed following the instructions in the tests manuals. Since the Denver-II has no raw score, we added items passed to items preceding the basal level, following general scoring principles. Equally, for the WHO-Motor we constructed the raw score adding all items the child passed. Hence, raw scores increased with age for all tests, except for the ASQ-3 which had age-specific questionnaires.

Neither the Bayley-III nor any of the short tests have been standardized for Colombia. Moreover, the Bayley-III composite scores were shown to vary by age in unusual ways in this sample [35], suggesting the unsuitability of the external norms (derived from a US representative sample). Therefore, after removing testers’/interviewers’ effects, we internally standardized the residuals of the raw scores over age using age-conditional means and standard deviations (SDs) using non-parametric methods (S1 Text). Unlike using norms from the reference populations for each test, this standardization method has the advantage that it handles age effects consistently across tests, thus facilitating comparisons.

For each test, the trainer recorded total administration time during the supervised assessments and computed the average. Test administration costs (i.e. purchase of the test kit and per child administration fees) were also documented. We examined internal consistency using Cronbach’s alpha (α) and test-retest reliability using *ICCs*. We investigated concurrent validity between the short tests and the Bayley-III using Pearson correlations (*r*) by domain and 12-months-of-age groups. All correlations used the internally standardized scores, which is equivalent to computing partial correlations controlling for testers/interviewers and age flexibly. *P* values for the correlations were computed using bootstrapping methods, with 1000 replications and clustering by age and sector [38]. We classified correlations as low (*r* = 0.20–0.39), moderate (*r* = 0.40–0.59), and high (*r* = 0.60–0.79) [39]. We next compared the correlations of each of the short tests with the Bayley-III using the same approach (i.e. bootstrapped *P* values). Finally, we further explored concurrent validity in all ages combined by computing Pearson correlations (*r*) between scores in each test and a set of variables theoretically related to child development—including maternal education, the household wealth index, and play activities and play materials in the home. As a robustness test, we computed canonical correlations (correlations amongst sets of variables—in this case, the scales of a test) to account for the fact that the measures analyzed are multi-dimensional. This also corrected for the large number of correlations being analyzed.

All statistical analyses were performed using Stata 13.1 (StataCorp, College Station, TX). The ethical committee at the Instituto de Ortopedia Infantil Roosevelt in Bogota reviewed the study protocols and considered them to be fully compliant with required ethical practice. Written informed consent to participate in the study was obtained from parents on behalf of the children enrolled. All analysis is performed on anonymous and de-identified data, with personal identifiable information being securely kept in a restricted-access drive.

## Results

Of the 1,533 children tested at home, 1,330 (86.8%) had a Bayley-III assessment. Of these, 4 (0.3%) did not complete the test and 15 (1.1%) scored <70 in any of the Bayley-III composite scales and were dropped (Fig 1). Children given batteries A and B were comparable in terms of their Bayley-III raw and composite scores, as well as in terms of their socio-economic characteristics (Table 2). The only significant difference between the two samples was father’s education (*P* = 0.026). The samples were also well-balanced by age group and gender. Mean composite scores of the Bayley-III were in the normal range, although Standard Deviations (SD) were low, further justifying the pertinence of the internal standardization. Internal consistency and test-retest reliabilities after 6–19 days were very good for the Bayley-III (*ICCs* ≥0.9) and higher than for any of the short tests (Table 1).

**Table 2:** Characteristics of Children in the Study Sample by Battery.

	Battery A (*n*_*A*_ = 676)	Battery B (*n*_*B*_ = 635)
Child's age, %		
6–18 months	33.7	33.9
19–30 months	33.6	35.7
31–42 months	32.7	30.4
Socio-economic sector (strata), %		
1. Lowest	30.3	29.8
2.	32.7	37.0
3.	36.1	32.6
4. Highest	0.9	0.6
Girls, %	47.6	51.0
Premature (gestional age <37 weeks), %	15.2	15.1
Birth weight^a^, gr, mean (SD)	3066 (514)	3015 (510)
Stunted (z-score height-for-age <-2SD)	16.9	17.7
Mother's age^a^, y, mean (SD)	27.2 (6.9)	26.6 (6.4)
Mother's education^a^, y, mean (SD)	10.3 (3.4)	10.4 (3.3)
Father's education^a^, y, mean (SD)	8.15 (4.0)	8.7 (4.0)
**Bayley-III Raw Scores, mean (SD)**		
Cognitive	58.7 (14.5)	58.8 (13.5)
Receptive language	25.3 (9.3)	25.6 (8.9)
Expressive language	25.0 (10.3)	25.5 (10.2)
Fine motor	39.5 (10.1)	39.1 (10.0)
Gross motor	52.2 (11.7)	52.6 (11.4)
**Bayley-III Composite Scores, mean (SD)**		
Cognitive	97.8 (7.8)	98.9 (9.9)
Language	95.3 (8.4)	97.8 (11.1)
Motor	99.3 (10.1)	99.7 (11.6)

^a^ Missing data for: birth weight (n_A_ = 638, n_B_ = 552), mother's age (n_A_ = 668, n_B_ = 618), mother's education (n_A_ = 674, n_B_ = 633), father's education (n_A_ = 639, n_B_ = 576). SD is Standard Deviation.

### Administration Time and Cost

The cost of the kit of test materials and per child administration fee was substantially higher in the Bayley-III than in any of the short tests (Table 1). The Bayley-III also took longer to administer and required more skill to learn and give. Of the multi-dimensional short tests, the BDI-2 took longest and was the most expensive. The Denver-II and ASQ-3 were intermediate both in terms of time and cost; and the single-domain tests were, as expected, the shortest and cheapest of the short tests. In fact, the WHO-Motor was free. Training time increased with the length of the test. Scales or tests relying mostly on maternal reports were the easiest to train.

### Internal Consistency and Reliability of the Short Tests

For the short tests, Cronbach’s α were generally good except for the ASQ-3 which had low values (α<0.6) in two scales (fine motor and personal-social) (Table 1). Test-retest reliabilities after 2–11 days were only available for 11 or 12 children but were generally satisfactory (*ICCs* ≥0.7). The only exceptions were the fine motor scale in the ASQ-3 and the gross motor and personal-social scales in the Denver-II, all with *ICCs* <0.53.

### Concurrent Validity

#### Same Domain Scales

**Fig 2:**
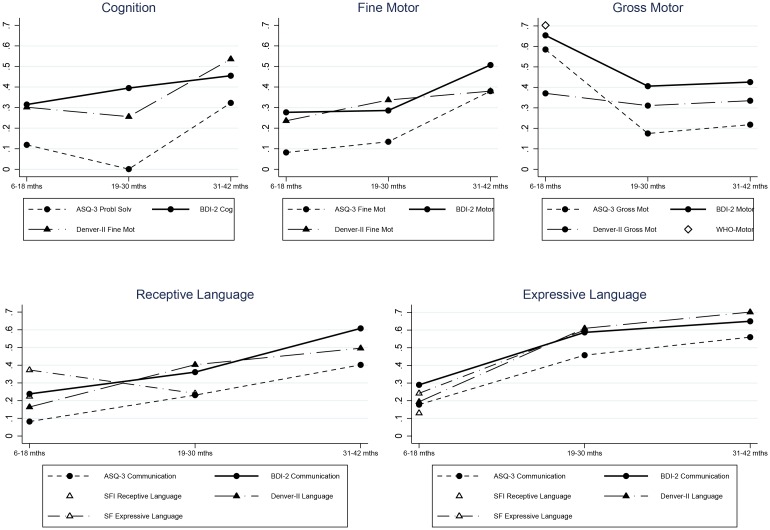
Concurrent Validity between the Bayley-III and the Short Tests by Matching Domain and Age Group.

Scales of the Bayley-III and the short tests measuring the same developmental domains were correlated by age groups (Fig 2). Given that the Denver-II did not have a cognitive scale, we correlated its fine motor-adaptive scale with cognition. Similarly, we correlated the communication/language scale in the multi-dimensional screeners with both the Bayley-III expressive and receptive language scales; and the BDI-2 motor scale with both the Bayley-III fine and gross motor scales. There was no matching Bayley-III scale for the personal-social or adaptive scales in the short tests.

**Cognitive, language, and fine motor:** The Denver-II and BDI-2 cognition/fine motor-adaptive, language/communication, and fine motor scales had similarly low but significant correlations with the corresponding Bayley-III scales at 6–18 months. Validity increased slightly at 19–30 months but only language reached moderate or high levels. Concurrence continued to improve over 30 months for all domains, language attaining the highest levels.

A comparison across the multi-dimensional tests showed that the ASQ-3 scales consistently had lower correlations with the Bayley-III than the Denver-II and the BDI-2. These correlations were significantly lower in 16 of 24 comparisons (*P*<0.05, Table 3). The only exception was the correlation between the ASQ-3 and the Bayley-III fine motor scales at 31–42 months, which was the same as that with the BDI-2. In the youngest group, the ASQ-3 correlations were generally trivial and non-significant. The ASQ-3 problem solving scale did not significantly predict Bayley-III cognition until over 31 months (Table 3).

**Table 3:** Correlations among Bayley-III Scales and between Scales in the Bayley-III and the Short Tests by Age Group.

	Bayley-III, 6–18 months					Bayley-III, 19–30 months					Bayley-III, 31–42 months				
	Cognitive	Receptive Language	Expressive Language	Fine Motor	Gross Motor	Cognitive	Receptive Language	Expressive Language	Fine Motor	Gross Motor	Cognitive	Receptive Language	Expressive Language	Fine Motor	Gross Motor
**Bayley-III**	*n* = 443					*n* = 454					*n* = 414				
Cognitive	1					1					1				
Receptive Language	0.437***	1				0.604***	1				0.590***	1			
Expressive Language	0.356***	0.502***	1			0.494***	0.554***	1			0.603***	0.639***	1		
Fine Motor	0.533***	0.490***	0.408***	1		0.525***	0.435***	0.377***	1		0.528***	0.458***	0.457***	1	
Gross Motor	0.333***	0.329***	0.232***	0.354***	1	0.392***	0.407***	0.350***	0.421***	1	0.381***	0.329***	0.334***	0.363***	1
**ASQ-3**	*n* = 221					*n* = 224					*n* = 219				
Problem Solving	**0.119**	0.010	0.071	0.062	0.026	**0.001**	0.075	0.133*	0.091	0.005	0.323***	0.374***	0.454***	0.292***	0.111
Communication	0.104	**0.082**	0.178**	0.142*	0.164*	0.141*	0.231***	0.458***	0.069	0.067	0.361***	0.402***	0.560***	0.286***	0.147*
Fine Motor	0.084	0.084	0.077	**0.082**	0.151*	0.135*	0.131*	0.171**	0.134*	0.176**	0.297***	0.256***	0.331***	0.380***	0.164*
Gross Motor	0.147*	0.148*	0.047	0.105	0.585***^b^	-0.053	0.013	0.025	0.010	0.175**	0.110	0.060	0.059	0.090	0.218***
Personal-Social	0.208**	0.184**	0.166*	0.208**	0.070	0.033	0.082	0.121	0.065	0.109	0.063	0.140*	0.240***	0.102	0.119
**Denver-II**	*n* = 225					*n* = 221					*n* = 212				
Language	0.206**	0.238***^a^	0.290***	0.187*	0.125	0.224***	0.361***^a^^,^^d^	0.587***^a^	0.111	0.175**	0.560***^e^	0.608***^a^	0.650***	0.443***	0.284***
Fine Motor-Adaptive	0.315***^a^	0.279***	0.168*	0.277***^a^	0.153*	0.395***^a^	0.339***	0.345***	0.286***^a^	0.257***	0.455***	0.377***	0.426***	0.507***^a^	0.229***
Gross Motor	0.264***	0.270***	0.085	0.180**	0.654***^b^	0.133*	0.171**	0.207***	0.198**	0.406***^a^	0.256***	0.263***	0.270***	0.239***	0.426***^a^
Personal-Social	0.366***	0.279***	0.296***	0.240***	0.244***	0.099	0.174**	0.185**	0.182**	0.197**	0.274***	0.194**	0.200**	0.166*	0.111
**BDI-2**	*n* = 215					*n* = 227					*n* = 193				
Cognitive	0.302***^a^	0.223***	0.209**	0.274***	0.244***	0.256***^a^	0.297***	0.327***	0.253***	0.146	0.536***^a^	0.444***	0.484***	0.404***	0.243***
Communication	0.205**	0.164*	0.194**	0.195**	0.229***	0.350***	0.403***^a^	0.610***^a^	0.196**	0.245***	0.488***	0.496***	0.702***^a^	0.329***	0.325***
Motor	0.147*	0.161*	0.109	0.236***	0.371***	0.288***	0.231***	0.268***	0.337***^a^	0.311***	0.379***	0.273**	0.386***	0.380***	0.335***
Personal-Social	0.025	0.072	0.057	0.001	0.077	0.145	0.193**	0.286***	0.019	-0.015	0.210**	0.226**	0.293***	0.110	0.126
Adaptive Skills	0.090	0.206**	0.228***	0.137*	0.271***	0.168*	0.218***	0.234***	0.241***	0.257***	0.201**	0.255***	0.240***	0.189*	0.077
**SFI & SFII**	*n* = 192^f^					*n* = 226									
Receptive Language	0.187**	0.224***	**0.130**	0.088	0.168*										
Expressive Language	0.258***	0.373***^a^^,^^b^^,^^c^	0.242***	0.204***	0.176*	0.168*	0.241***	0.600***^a^	0.077	0.134*					
**WHO-Motor**	*n* = 152^g^														
Gross Motor	0.224**	0.126	0.282***	0.061	0.703***^b^										

Pearson correlations on internally standardised scores, Standard Errors (SE) computed using bootstrap stratifying by age category and socio-economic sector (*n* = 1000 replications):

* p<0.05,

** p<0.01,

*** p<0.001. Matching scales bolded.

^a^ Concurrence larger than ASQ concurrence, matching domain;

^b^ Concurrence larger than BDI-2 concurrence, matching domain;

^c^ Concurrence larger than Denver-II concurrence, matching domain;

^d^ Concurrence larger than SFII concurrence, matching domain;

^e^ Concurrence larger than Denver-II fine motor-adaptive concurrence with Bayley-III cognitive scale.

^f^ Children 8–18 months;

^g^ Children 6–15 months.

The SFI expressive language scale had slightly higher correlations with both Bayley-III language scales than the SFI receptive, although the difference was not statistically significant. In the youngest group, the SFI expressive scale had a low correlation with Bayley-III receptive language. Even so, this correlation was significantly larger than that with the Denver-II and BDI-2 language/communication scales (both *P*<0.05) and with the ASQ-3 communication scale (*P* <0.001). The correlation of the SFI expressive scale with the Bayley-III expressive language scale was however similar to that with the other short tests. At 19–30 months, the SFII expressive had a low correlation with the Bayley-III receptive language, which was significantly lower than the Denver-II (*P*<0.05). Nevertheless, the SFII expressive had a high correlation with the Bayley-III expressive language scale, similar to the Denver-II and the BDI-2, and significantly higher than the ASQ-3 (*P*<0.05).

**Gross motor:** Gross motor scales behaved differently from the other domains. The BDI-2 motor scale showed low correlations with the Bayley-III gross motor that changed little throughout the age range (Fig 2). The Denver-II and ASQ-3 gross motor correlations were moderate to high for children 6–18 months (significantly larger from the BDI-2, *P*<0.05) and then decreased for older children. The Denver-II concurrence fell to moderate levels and the ASQ-3 fell to low levels, which were significantly lower than those for the Denver-II (*P*<0.05). For children 6–15 months, the WHO-Motor had a high correlation with the Bayley-III gross motor. This correlation was higher than any other test for gross motor development, albeit only significantly higher from the BDI-2 (*P*<0.001).

#### Different Domains Scales

Occasionally, correlations between the Bayley-III and the short tests were higher between scales measuring different functions than those between scales measuring the same functions (Table 3). This happened less frequently as the children aged. In the youngest group, the personal-social scales of the Denver-II and ASQ-3 correlated with cognition, language, and fine motor. For children over 18 months, the language scales were often significantly related to the Bayley-III cognition. Over 30 months, the Denver-II language scale correlation with cognition was significantly higher than the fine motor-adaptive scale (p<0.05). There were few other clear patterns in the cross-correlations.

The domain-specific tests also correlated with other domains. In the youngest group, the SFI expressive language had significant but low correlations with cognition and fine motor, and the WHO-Motor had low but significant correlations with cognition and expressive language.

### Correlations with Other Variables

In the combined age groups, all scales in the Bayley-III were significantly correlated with maternal education, household wealth, play activities and play materials, except for gross motor which had very low significant correlations with maternal education and play materials only (Table 4). Regarding the short tests, most of the scales showed low but significant correlations with at least two social background factors. The exceptions were all gross motor scales, as well as the expressive language scale in the SFI in children under 18 months and the personal-social scale in the Denver-II. The BDI-2 showed the highest correlations, but they tended to be smaller than those observed for the Bayley-III.

**Table 4:** Correlations of the Bayley-III and the Short Tests with Maternal Education, Household Wealth, Play Activities and Play Materials in the Home, All Ages Combined.

	Maternal Education	Wealth Index	Play Activities	Play Materials
**Bayley-III** (*n* = 1311)				
Cognition	0.210***	0.235***	0.189***	0.271***
Receptive Language	0.216***	0.191***	0.214***	0.248***
Expressive Language	0.206***	0.224***	0.209***	0.243***
Fine Motor	0.124***	0.145***	0.119***	0.179***
Gross Motor	0.079**	0.034	0.023	0.056*
**ASQ-3** (*n* = 664)				
Problem Solving	0.127**	0.071	0.176***	0.177***
Communication	0.142***	0.136***	0.222***	0.156***
Fine Motor	0.063	0.067	0.167***	0.133***
Gross Motor	-0.025	0.046	0.069	0.019
Personal-Social	0.019	0.034	0.152***	0.088*
**Denver-II** (*n* = 658)				
Language	0.170***	0.165***	0.184***	0.173***
Fine Motor-Adaptive	0.102**	0.121**	0.097*	0.109**
Gross Motor	0.022	0.020	-0.021	-0.011
Personal-Social	-0.034	-0.019	0.064	0.010
**BDI-2** (*n* = 635)				
Cognitive	0.202***	0.173***	0.164***	0.181***
Communication	0.210***	0.176***	0.224***	0.245***
Motor	0.139***	0.163***	0.135***	0.179***
Personal-Social	0.144***	0.136***	0.240***	0.231***
Adaptive Skills	0.074	0.094*	0.276***	0.193***
**SFI** (*n* = 192)^a^				
Receptive Language	0.147*	0.127	0.267***	0.251***
Expressive Language	0.040	-0.060	-0.007	-0.005
**SFII** (n = 226)^b^				
Expressive Language	0.136*	0.094	0.229***	0.200**
**WHO-Motor** (*n* = 152)^c^				
Gross Motor	-0.036	0.008	0.082	0.018

* p<0.05,

** p<0.01,

*** p<0.001.

^a^ Children 8–18 months.

^b^ Children 19–30 months.

^c^ Children 6–15 months.

For robustness, all analyses were repeated using the composite Bayley-III scores and the ASQ-3 scores computed using the original six-item questionnaires; and we further divided the sample by 6-months-of-age groups. Results were little altered. Canonical correlations results indicated that the scales showing the highest Pearson correlations by pair of tests and age group were those that significantly contributed to the canonical variates. Correlations amongst canonical variates (canonical correlations) displayed the same pattern of results as the one observed using Pearson (bivariate) correlations and described above—the magnitude of the correlation being similar and often higher (S1 Table).

## Discussion

We examined the use of three multi-dimensional screeners and two single-domain tests of child development that have previously been used in large scale studies in LMICs. The internal reliability was generally good or acceptable, except for the ASQ-3, and all tests correlated with socio-economic variables as theoretically expected.

In the multi-dimensional tests, concurrent validity with the Bayley-III varied by age and domain. The language, cognitive, and fine motor scales of the Denver-II and BDI-2 had low but significant validity below 19 months, moderate at 19–30 months, and moderate-to-high over 30 months. Language generally showed the highest levels over 19 months. The ASQ-3 had poorer validity in these scales than the other two tests throughout the age range and was trivial under 19 months. The gross motor scales behaved differently: they had high validity below 19 months, which then declined. The BDI-2 was an exception probably because it combined fine and gross motor and had low correlations with the Bayley-III gross motor at all ages.

Regarding the single-domain tests, the WHO-Motor had high concurrence with the Bayley-III gross motor scale up to age 15 months. The SFI receptive language scale, only available under 19 months, had slightly lower correlations than the SFI expressive with both Bayley-III language scales. It is possible that mothers found it easier to report words used than words understood. Expressive language had low correlations with the Bayley-III language scales under 19 months, and high correlations between 19–30 months.

### Choice of test

The choice of tests depends on the availability of time, funds and qualified testers, all of which are usually limited in large surveys. The choice also depends on test validity, the amount of adaptation required, age of the children and study objectives. The main outcomes of interest, for example, may vary depending on whether the aim is to establish the broad developmental profile of a population or to evaluate an intervention, as well as on the type of intervention being evaluated.

All the multi-dimensional tests spread over the entire age range and the concurrent validity of the cognitive, language, and fine motor scales was little different between the BDI-2 and the Denver-II. However, the BDI-2 was much longer to administer, expensive, and required the most training. The Denver-II and the ASQ-3 took less time to give and required similar amounts of materials. However, the ASQ-3 had lower concurrent validity, suggesting that the Denver-II was the most suitable for use at-scale. In Nicaragua, the Denver-II, administered at home, was sensitive to the impact of a cash transfer program [19]. The poor validity of the ASQ-3 below 30 months is concerning given the test is increasingly used in large scale studies [18, 40]. It is possible that the language modifications to Spanish may have changed the psychometrics of the test. In any event, these findings do not concern its validity as a screener of high risk children.

The single-domain tests were the most feasible to give, being short, inexpensive, and requiring little training. Whilst, their age range is limited, they offered reasonable levels of concurrence for the domains and ages for which they are available. Therefore, they might be of consideration for survey work. The WHO-Motor was highly valid for gross motor development under 16 months, and had low correlations with cognition and expressive language. This concurs with findings from the Bangladeshi study discussed above [21]. However, monthly assessments were used in Bangladesh and may be more accurate than one examination only, as in the present study.

Similarly, the SFI and SFII expressive had at least as good validity as the language scales of the multi-dimensional tests and low correlations with cognition and fine motor under 19 months in Bogota. In Bangladesh, vocabulary reports locally developed from the SFII also had moderate concurrent validity (*r* = 0.41, P<0.01) with the Bayley-II MDI at 18 months, and predicted IQ at 5 years (*r* = 0.37, *P* <0.01) [22]. An advantage of maternal reports of early vocabulary is that disadvantaged young children, who tend to be inhibited in LMICs, do not have to speak to the tester. A disadvantage is that a new inventory has to be ‘developed’ for every new language, which is time consuming and requires skill. In addition, some adaptations may be needed when using the same language in different countries/contexts. Nonetheless, this is feasible and in fact the SFs are already available in many languages [41].

It is generally agreed that multi-dimensional tests are most desirable [14]. But, where resources are limited, it may be possible to use selected scales of a test or single-domain tests, depending on the children’s age and purpose of the survey. For example, to evaluate psychosocial stimulation programs that rarely benefit gross motor development, the language and fine motor-adaptive Denver-II scales could be used. For nutritional interventions, however, which more often affect motor development in younger children, the WHO-Motor would be useful in children under 16 months; especially since it has low but significant correlations with the cognitive and expressive language scales. If language is the focus of interest and children are under 30 months, then the SFI and SFII could be used without the receptive scale.

Overall, the low-to-moderate concurrent validity of all tests except the gross motor scales in the youngest children concurs with reported difficulties in assessing young children’s development, particularly at large scale [10, 13, 14]. As a result, with the exception of gross motor, all other tests had limited validity under 18 months. However, examination of predictive validity is needed to be certain and to complement these findings since concurrent and predictive validity may not necessarily be closely related. For example, the Bangladeshi language test had moderate concurrent validity with the Bayley-II at 18 months but similar predictive validity of later IQ [22]. Similarly, more research on developing or modifying tests for children under 24 months would be desirable.

### Study Limitations and Strengths

A limitation of the study is that cross-sectional data prevents investigation of predictive validity. We are currently preparing a long term follow-up after 5.5 years, when the study participants will be 6–9 years, to examine it. Another limitation is that the Bayley-III was not standardized in Colombia. Nonetheless, it showed good internal and test-retest reliability and was related to socio-economic characteristics as expected. We previously reported that the scores showed differences by wealth quartiles from the first year of life that increased to 42 months [35]. Furthermore, the correlations of the Bayley-III scales among each other were similar to those reported in the test manual [12]. The scales also showed acceptable levels of predictive validity with measures of cognition, language, and school readiness at age 5 in a contemporaneous Colombian study by the same researchers. These findings strongly suggest that the Bayley-III is valid in this population and an appropriate ‘gold standard’. The study strengths are the large, population-based sample of children 6–42 months, and the quality of our ‘gold standard’.

## Conclusions

Measuring ECD outcomes for very young children at-scale is challenging. However, multi-dimensional screeners and single-domain tests offer feasible, reliable alternatives. Concurrent validity varies by domain and age. The scales with the highest concurrence were gross motor under 19 months and language above. The Denver-II was the most feasible and valid multi-dimensional test and the ASQ-3 generally performed poorly under 31 months. Investigation of predictive validity and sensitivity to interventions is needed to further support these findings, which should be helpful in the selection of instruments and design of future large scale studies interested in the measurement of child development.

## Supporting Information

S1 Table Canonical Correlations between Bayley-III and Short Tests Canonical Covariates, by Age Group. (DOCX)

S1 Text Internal Standardization of Scores using Age-Conditional Means and SDs. (DOCX)
