# Supplementary material for: Concurrent Validity and Feasibility of Short Tests Currently Used to Measure Early Childhood Development in Large Scale Studies
Source: PLoS One. 2016 Aug 22;11(8):e0160962. doi: 10.1371/journal.pone.0160962 (PMC4993374; doi:10.1371/journal.pone.0160962)
Supplement: S1 Table — (DOCX) [file pone.0160962.s001.docx]

**S1 Table. Canonical Correlations among Bayley-III and Short Tests Canonical Covariates, by Age Group.**

Correlations between canonical variates whose statistically significant raw coefficients are the scales corresponding to the cells where the correlation is reported: * p<0.05, ** p<0.01, *** p<0.001. Matching scales bolded.

+ Domains/scales that also significantly contribute to the canonical variates. ^a^ Children 8-18 months; ^b^ Children 6-15 months.
