# Supplementary material for: Concurrent Validity and Feasibility of Short Tests Currently Used to Measure Early Childhood Development in Large Scale Studies
Source: PLoS One. 2016 Aug 22;11(8):e0160962. doi: 10.1371/journal.pone.0160962 (PMC4993374; doi:10.1371/journal.pone.0160962)
Supplement: S1 Text — (DOCX) [file pone.0160962.s002.docx]

**S1 Text. Internal Standardization of Scores using Age-Conditional Means and SDs.**

For each scale, we removed tester effects from the raw score by running a regression of the raw scores on tester dummies using Ordinary Least Squares (OLS). We constructed the residuals of these regressions, which we standardized by age using non-parametric methods as follows. First, we computed the age-conditional mean using the fitted values of the regression in (1), estimated by kernel-weighted local polynomial smoothing methods:

$Y_{i}=f(X_{i})+\varepsilon_{i} \forall i$ (1)

where $Y_{i}$ is the residual of the raw score of child *i* in a given scale of a regression on tester dummies. $X_{i}$ is the age of the child in days. Next, we regressed the square of the residuals in (1) on age of the child (in days) as shown in the kernel-weighted local polynomial regression in (2):

${(Y_{i}-\hat{f}_{i})}^{2}=g(X_{i})+v_{i} \forall i$ (2)

Our estimate of the age-conditional standard deviation (SD) is the square root of the fitted values $\hat{g}_{i}$ in (2). Finally, we computed the internally age-adjusted z-score, $ZY_{i}$, by subtracting from the residual of the raw score the within sample age-conditional mean estimated in (1) and dividing by the within sample age-conditional SD obtained from (2). More specifically:

$ZY_{i}=\frac{Y_{i}-\hat{f}_{i}}{\sqrt{\hat{g}_{i}}} \forall i$ (3)

This resulted in smooth normally distributed internally standardized scores, with mean zero across the age range.
